# Common Genetic Origins for EEG, Alcoholism and Anxiety: The Role of *CRH-BP*

**DOI:** 10.1371/journal.pone.0003620

**Published:** 2008-10-31

**Authors:** Mary-Anne Enoch, Pei-Hong Shen, Francesca Ducci, Qiaoping Yuan, Jixia Liu, Kenneth V. White, Bernard Albaugh, Colin A. Hodgkinson, David Goldman

**Affiliations:** 1 Laboratory of Neurogenetics, National Institute on Alcohol Abuse and Alcoholism, National Institutes of Health, Bethesda, Maryland, United States of America; 2 Center for Human Behavior Studies, Inc, Weatherford, Oklahoma, United States of America; Vrije Universiteit Amsterdam, Netherlands

## Abstract

The resting EEG is a dynamic index of cortical activation, cognitive function and consciousness and is therefore an intermediate phenotype for many behaviors in which arousal is implicated such as anxiety and alcoholism. We performed a dense whole genome linkage scan using 3878 unlinked SNPs in a large pedigree derived from a population isolate sample of 328 Plains American Indians. Alpha (8–13 Hz), theta (4–8 Hz) and beta (13–30 Hz) EEG power was heritable (0.58–0.27) and stable over a 2 year period (r = 0.82–0.53). Genetic correlations between frequency bands were high (0.75). Linkage peaks for EEG power in all three frequency bands converged on chromosome 5q13-14 with genome-wide significant LOD scores of 3.5 (empirical p<0.0001) for alpha and beta power. A logical candidate gene, corticotropin releasing hormone-binding protein (*CRH-BP*), was located at the apex of these convergent linkage peaks. *CRH-BP* was significantly associated with alpha power in the Plains Indians and also in a replication sample of 188 Caucasians. Moreover, the same SNPs and haplotypes, located within the *CRH-BP* haplotype block, were also associated with anxiety disorders in the Plains Indians and alcohol use disorders in the Caucasians. *CRH-BP* modulates CRH which influences cortical and hippocampal EEG activity and is the primary mediator of the neuroendocrine stress response. Our results suggest a likely role for *CRH-BP* in stress-related alcoholism and highlight the use of the resting EEG as an intermediate phenotype for arousal-related behaviors such as anxiety and addiction.

## Introduction

The electroencephalogram (EEG) is a recording of the rhythmical, electrical activity of the brain that is thought to derive from extracellular current flow associated with summated postsynaptic potentials in synchronously activated, pyramidal cells that are perpendicular to the cortical surface [1], [2]. The EEG pattern is constantly changing depending on mental activity, relaxation, drowsiness and sleep and this dynamic process is therefore an index of cortical activation, cognitive function and consciousness. The EEG may thus be considered an intermediate phenotype for complex behaviors and psychopathology in which arousal is implicated, such as anxiety, depression and addiction.

In the healthy awake adult two rhythms, alpha (8–13 Hz) and beta (13–30 Hz) dominate the resting EEG. Lower frequency rhythms: theta (4–8 Hz) and delta (0.1–4 Hz) are less prominent [2]–[4]. The alpha rhythm is maximal posteriorly under conditions of eyes-closed relaxation and mental inactivity. Higher alertness attenuates or suppresses the alpha rhythm which is then supplanted by desynchronized low voltage fast activity. However, alpha has also been implicated in information processing in a variety of cognitive tasks [5]. Beta, a lower voltage, irregular waveform has a diffuse distribution and predominates in mentally active individuals. The theta frequency is important in infancy, childhood, drowsiness and sleep and is the dominant rhythm in non-primates. Theta oscillations represent the ‘on-line’ state of the hippocampus and are thought to be implicated in memory and learning [6]. The delta frequency is commonly seen in infants and deep sleep in adults. The EEG can be characterized by amplitude and frequency but for research purposes the recording is usually transformed to yield spectral power per frequency band.

Some studies have shown that decreased alpha EEG power and increased theta and beta EEG power is associated with psychopathology, particularly addiction, although no one study has investigated disease associations with all three frequency bands together. A family history of alcoholism predicts reduced alpha power [7]. Low voltage alpha EEG, a distinct phenotype [8] has been shown to be more common in alcoholics with anxiety disorders [9]. Increased beta activity has been associated with alcoholism in men [10], a family history of alcoholism [11], [12] and relapse in abstinent alcoholics [13]. Alcoholics have been shown to have increased theta power [14].

Although there is considerable inter-individual variability, the distinctive resting EEG pattern of an individual tends to be stable throughout healthy adulthood [8]. Test-retest reliability for resting EEG power is high (r = 0.8–0.9) [15]–[17]. The heritability of eyes closed, resting EEG power using monopolar references (as in our study) tends to be the same at all scalp locations [17]–[19] and has been shown to be approximately 80–85% for theta, alpha and beta and 40% for delta in twin studies [18]–[22] and 23–68% in one large family based study [23]. The same genes appear to influence alpha power throughout the brain [18]. Genetic correlations between alpha, beta, theta and delta power (0.55 to 0.75) indicate that a substantial portion of the genetic variance can be attributed to a common source [17], however frequency band-specific and region-specific genetic factors are also influential [19].

The evidence for both shared and specific genetic influences on the resting EEG is compelling. So far, only one study has identified a genetic association. Using the Collaborative Study on the Genetics of Alcoholism (COGA) dataset, significant linkage for beta EEG power was reported for the region of the chromosome (chr) 4 GABA_A_ receptor cluster [24]. This linkage was subsequently tracked to the *GABRA2* gene [25].

Because the resting EEG is an indicator of cortical activation it may be regarded as an intermediate phenotype for anxiety, stress and addiction. Therefore candidate genes for the resting EEG are likely to encompass not only genes directly implicated in current flow and synchronization as well as rhythm generation, but also genes influencing arousal-related behavior. In our study we performed a dense whole genome linkage scan in Plains American Indians, a population isolate, who derived predominantly from one large pedigree. Our linkage scan utilized nearly 4000 unlinked single nucleotide polymorphisms (SNPs) at an average distance of 1 cM. The aim of our study was to identify shared and specific locations of genetic effects on alpha, beta and theta resting EEG power and to identify candidate genes in these regions. In secondary analyses, association between EEG power and candidate genes in the Plains Indian sample would be tested in a sample of U.S. Caucasians in whom resting EEG had also been recorded.

## Results

The resting EEG was recorded from six scalp electrodes: FZ (frontal-central), P3, PZ, P4 (parietal central, left and right), and O1 and O2 (occipital left and right). All results are for eyes closed, normalized (log_10_ transformed) resting EEG power. EEG data was available for 364 Plains Indians.

There was no relationship between alpha or beta EEG power and alcohol use disorders (AUD) or anxiety in this population that has a high prevalence of lifetime AUD (46% in women, 75% in men). In the Plains Indians without anxiety disorders, theta power at FZ was significantly lower in alcoholics compared with non-alcoholics (p = 0.0301).

### Stability of resting EEG power

EEGs were recorded under the same paradigm in 43 unrelated individuals retested after an interval of two years. The correlations between these two time points for EEG power at PZ were: alpha: r = 0.82, p<0.0001; beta: r = 0.55, p = 0.0001; theta: r = 0.53, p = 0.0003 (all 1df).

### EEG power: phenotype – phenotype correlations

As can be seen from Table 1, frontal theta power was significantly correlated with parietal and occipital theta power (r = 0.60 to 0.70). Likewise, frontal alpha (r = 0.63–0.70) and beta power (r = 0.70–0.82) was significantly correlated with alpha and beta power respectively at parietal and occipital scalp locations. Theta, alpha and beta power was highly correlated with theta, alpha and beta power respectively between parietal and occipital locations (r = 0.80–0.91). All results were significant (p<0.0001, 1df).

EEG power in the different frequency bands was moderately and positively correlated (p<0.0001, 1df): for example, at PZ: theta vs alpha, r = 0.60; theta vs beta, r = 0.58; alpha vs beta, r = 0.57; at FZ: theta vs alpha, r = 0.78; theta vs beta, r = 0.54; alpha vs beta, r = 0.62.

EEG power in the different frequency bands was positively correlated to the same degree within alcoholics as well as non-alcoholics.

**Table 1:** Correlations between theta, alpha and beta EEG spectral power at frontal, parietal and occipital electrode sites.

		FZ			PZ			O1			O2		
		θ	α	β	θ	α	β	θ	α	β	θ	α	β
FZ	θ		0.78	0.54	**0.70**	0.44	0.33	**0.60**	0.44	0.29	**0.62**	0.46	0.28
	α			0.62	0.52	**0.70**	0.42	0.44	**0.63**	0.32	0.43	**0.64**	0.32
	β				0.53	0.49	**0.82**	0.53	0.55	**0.70**	0.52	0.56	**0.72**
PZ	θ					0.60	0.58	**0.86**	0.59	0.49	**0.90**	0.63	0.51
	α						0.57	0.45	**0.80**	0.40	0.45	**0.81**	0.41
	β							0.59	0.65	**0.86**	0.58	0.65	**0.90**
O1	θ								0.66	0.64	**0.91**	0.60	0.59
	α									0.67	0.56	**0.91**	0.60
	β										0.54	0.58	**0.90**
O2	θ											0.64	0.61
	α												0.64
	β												

Electrodes: FZ: frontal central; PZ: parietal-central; O1, O2: occipital left and right.

Figures are values for ‘r’. All correlations are significant at p<0.0001.

Figures in bold indicate correlations between power in the same frequency band (theta (θ) 3–8 Hz; alpha (α) 8–13 Hz; beta (β) 13–30 Hz) at different locations.

EEG data was available for 364 participants.

### Heritability of EEG power

Heritability was highest for alpha power (0.38–0.58) followed by theta (0.28–0.40) and then beta (0.27–0.33) and did not differ between frontal and posterior locations for each frequency band (Table 2). There was a tendency for theta and alpha power to be more heritable in the left hemisphere than the right.

**Table 2:** Heritability of resting EEG spectral power at different scalp locations.

EEG freq band	EEG Electrode Location					
	FZ	P3	PZ	P4	O1	O2
Theta (3–8 Hz)	0.39 (0.12)	0.37 (0.13)	0.37 (0.13)	0.28 (0.13)	0.40 (0.12)	0.31 (0.12)
P value	5×10^−5^	4×10^−4^	5×10^−4^	5×10^−3^	4×10^−5^	0.002
Alpha (8–13 Hz)	0.58 (0.13)	0.56 (0.13)	0.54 (0.13)	0.38 (0.13)	0.43 (0.12)	0.47 (0.13)
P value	4×10^−8^	1×10^−7^	1×10^−7^	1×10^−4^	1×10^−5^	1×10^−5^
Beta (13–30 Hz)	0.27 (0.11)	0.30 (0.13)	0.33 (0.13)	0.30 (0.13)	0.29 (0.12)	0.27 (0.12)
P value	0.002	0.004	0.001	0.003	0.003	0.005

Heritability (S.E.) and P values are given for different electrode locations.

Heritability was computed using Z scores of log_10_ absolute EEG spectral power.

Electrodes: FZ: frontal central; PZ, P3, P4: parietal-central, left and right; O1, O2: occipital left and right.

### Genetic/environmental correlations at PZ of power in the three frequency bands

The genetic correlations were as follows: alpha and theta: 0.72 (S.E. 0.12); alpha and beta: 0.75 (S.E. 0.12); theta and beta: 0.72 (S.E. 0.18). The environmental correlations were: alpha and theta: 0.55 (S.E. 0.10); alpha and beta: 0.47 (S.E. 0.11); theta and beta: 0.51 (S.E. 0.10).

### Results from the whole genome linkage scan

We performed multipoint variance component linkage analyses using SOLAR with 3878 unlinked single nucleotide polymorphisms (SNPs) spaced at an average interval of 1 cM. Genotypes and EEG data were available for 266 Plains Indians, mostly from one large pedigree (n = 1004).

#### Genomewide significant linkage (LOD≥3.3)

**Figure 1:**
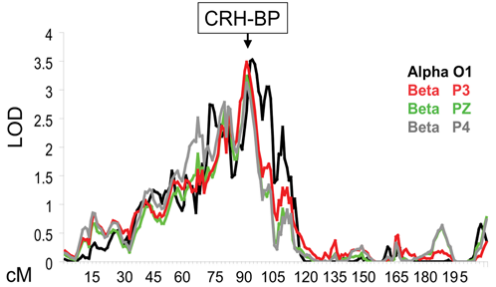
Chromosome 5q13-14: Convergence of Linkage Peaks for Alpha (8–13 Hz) and Beta (13–30 Hz) EEG power. Only linkage peaks with LOD scores>3.0 are shown here. *CRH-BP* = corticotropin releasing hormone-binding protein gene. O1 = occipital electrode; P3, PZ, P4 =  parietal electrodes.

We found a convergence of linkage peaks for alpha, beta and theta EEG power on chr 5 at 90–93 cM (Table 3, Figure 1). The linkage peak for alpha power at O1 had a logarithm of the odds (LOD) score of 3.53 (empirical p<0.0001), and the linkage peak for beta power had a maximum LOD score of 3.48 (empirical p<0.0001) at the three parietal leads. Theta power also had a linkage peak in this region with a maximum LOD score of 2.2 at PZ and O1 (Table 3).

**Table 3:** Whole genome linkage scan: LOD scores≥2.0 for normalized resting EEG spectral power.

EEG frequency band	Electrode location	Chromosome 4	Chromosome 5	Chromosome 22
		LOD [cM]	LOD [cM]	LOD [cM]
Theta	FZ			
3–8 Hz	P3			2.2 [2; 0–27]
	PZ		2.2 [79; 71–82]	2.4 [4; 0–26]
	P4			2.3 [2; 0–27]
	O1	2.5 [40; 38–43]	2.2 [76; 73–83]	**3.2** ^2^ [20; 4–26]
	O2			2.6 [2; 0–27]
Alpha	FZ		2.7 [79; 72–86]	
8–13 Hz	P3			
	PZ			
	P4		2.7 [79; 71–82]	
	O1		**3.5** ^1^ [93; 66–114]	
	O2	2.4 [48; 46–50]	2.6 [79; 66–99]	
Beta	FZ			
13–30 Hz	P3		**3.5** ^1^ [90; 52–103]	
	PZ		**3.2** ^2^ [90; 53–102]	
	P4		**3.1** ^3^ [90; 53–102]	
	O1		2.8 [90; 74–113]	
	O2		2.4 [90; 77–96]	

Electrodes: FZ: frontal central; PZ, P3, P4: parietal-central, left and right; O1, O2: occipital left and right.

Figures in parentheses are the centimorgan [cM] position of the apex and range of the peak ≥LOD = 2.0.

Empirical p values computed by 10,000 simulations using SOLAR: ^1^ = p<10^−4^; ^2^ = p = 10^−4^; ^3^ = p<10^−3^.

##### Candidate genes

We looked at the region of the peak with a LOD score of ≥2.0 (61–108 cM; 38–95 Mbp). Out of the 346 genes in this region we identified four candidate genes: corticotropin releasing hormone-binding protein (*CRH-BP,* MIM 122559) at 90 cM, homer homolog 1 (*HOMER 1,* MIM 604798) at 94 cM, cocaine-and amphetamine-regulated transcript (*CART,* MIM 602606) at 83 cM and serotonin receptor 1A (*HTR1A,* MIM 109760) at 77 cM. There were 20 genes located at the apex of the alpha and beta linkage peaks with LOD scores of 3.5 (90–93 cM, 75.7–77.6 Mbp). Of these, only *CRH-BP* could be considered a logical candidate gene for EEG and arousal-related behaviors.

#### Suggestive Linkage (LOD≥2.0)

We found suggestive evidence for linkage that was specific to particular EEG frequency bands: theta on chr 22 (LOD = 3.2) and on chr 4 (LOD = 2.5); alpha on chr 4 (LOD = 2.4) and on chr 11 (LOD = 2.2) and beta on chr 10 (LOD = 2.5) and on chr 2 (LOD = 2.1) (see supporting Materials S1 for further details).

### Candidate gene association analysis

#### CRH-BP

We chose to analyze results for *CRH-BP* since it was located at the apex of the convergent linkage peaks for alpha, beta and theta EEG power and because it is a logical candidate gene (see discussion). The *CRH-BP* genotypes (8 SNPs) for both Plains Indians and the U.S. Caucasian replication sample were available from our addictions array (described in Methods).

##### CRH-BP haplotype block structure

**Figure 2:**
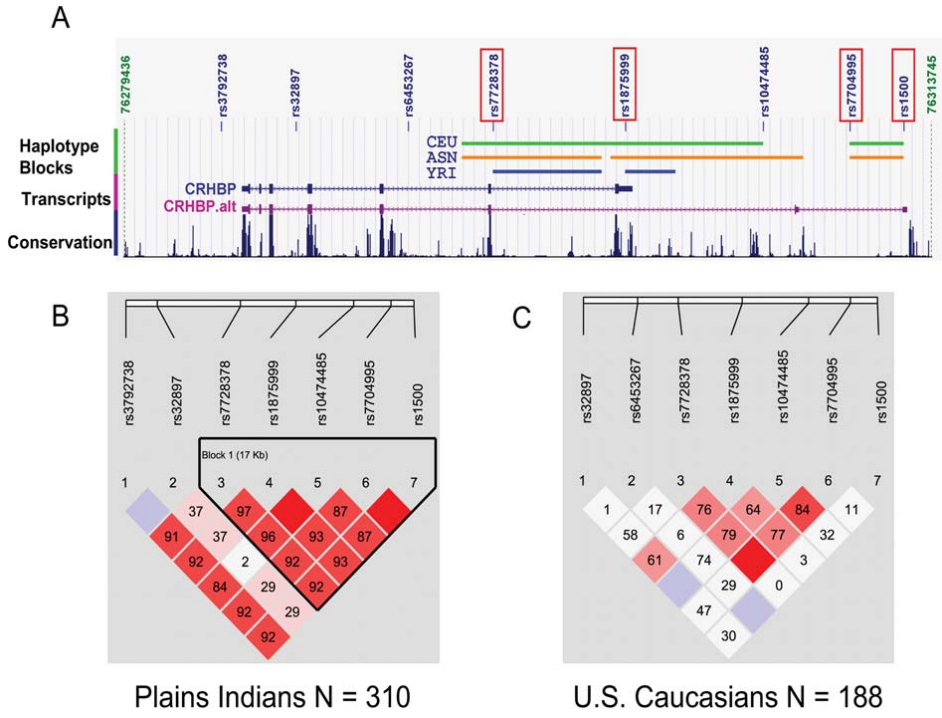
CRH-BP Gene Map and Haplotype Block Structure in Plains Indians and U.S. Caucasians. *CRH-BP*: corticotropin releasing hormone-binding protein gene. 2A: The 7 *CRH-BP* exons are indicated by rectangles. An alternative isoform (*CRH-BP* alt) expressed in the brain has exon 7 spliced out. The positions of the genotyped SNPs are indicated and the 4 SNPs with significant associations with EEG power are highlighted. The haplotype block structures of the three HapMap populations: CEU (Caucasian); YRI (African) and ASN (Chinese/Japanese) are provided. Conservation across 17 vertebrate species, from zebrafish to humans is indicated (www.genome.ucsc.edu). 2B, 2C: haplotype block structure across the two populations. The numbers are D′ values (linkage disequilibrium between SNP pairs).

There was one haplotype block in both the Plains Indians and the U.S. Caucasians incorporating the distal part of the gene. A similar pattern is seen in the HapMap (http://www.hapmap.org/) Caucasian and Asian populations (Figure 2A).

##### CRH-BP single SNP analyses: alpha EEG power

There was a significant genotype association with EEG alpha power together with a non-significant trend in the same direction for an association with beta and theta power. The association was greatest across all parietal leads with a trend in the same direction in the frontal and occipital leads. Results are presented for PZ, the electrode where alpha power is maximal in Plains Indians and where the phenotype–genotype association was the strongest.

In the Plains Indians, four SNPs: rs7728378, rs1875999, rs7704995 and rs1500, all in allelic identity and located within the haplotype block (Figure 2A), were significantly associated with alpha power (Table 4). The maximum effect size (converting back from log to absolute power) was 1.8. The significant association between rs7704995 genotype and alpha power (homozygote 11 vs homozygote 22, p = 0.007) was confirmed by the empirical p value derived from 10,000 simulations using SIMPED (p = 0.027).

**Table 4:** *CRH-BP* genotypes in Plains Indians: association with resting EEG alpha power and anxiety disorders.

SNPs	MAF	Alpha EEG power (µv^2^) at PZ					Anxiety vs Non-Anxiety			
		*CRH-BP* Genotypes			P values		Allele 1 freq		P values	
		11	12	22	Genotype	11 v 22	Anx	Non-Anx	Genotype	Allele
					2df	1df			2df	1df
rs3792738	0.12	4.25 (0.4)	4.31 (0.3)	4.51 (0.3)	0.15	0.10	0.83	0.88	0.42	0.20
N		239	65	6						
rs32897	0.07	4.24	4.23 (0.4)	4.27 (0.4)	0.75	0.93	0.09	0.07	0.74	0.59
N		1	44	265						
rs7728378	0.25	4.51 (0.3)	4.27 (0.4)	4.25 (0.4)	**0.02**	**0.005**	0.35	0.23	0.053	**0.016**
N		20	112	178						
rs1875999	0.24	4.51 (0.3)	4.27 (0.4)	4.25 (0.4)	**0.02**	**0.005**	0.35	0.23	**0.042**	**0.011**
N		20	109	181						
rs10474485	0.15	4.25 (0.4)	4.32 (0.4)	4.49 (0.3)	0.08	0.053	0.77	0.86	0.095	**0.031**
N		228	72	10						
rs7704995	0.23	4.51 (0.3)	4.27 (0.4)	4.25 (0.4)	**0.02**	**0.007**	0.34	0.21	**0.031**	**0.009**
N		18	105	187						
rs1500	0.23	4.51 (0.3)	4.26 (0.4)	4.25 (0.4)	**0.02**	**0.007**	0.34	0.21	**0.033**	**0.010**
N		18	106	186						

The table shows mean (S.D.) alpha power for individuals with each genotype (11, 12, 22). P values are derived from ANOVA; alpha power variation between genotypes (2 df) and between homozygotes (1df). Alpha power: log_10_ of absolute spectral power (µv^2^) in 8–13 Hz frequency band. Alpha power was maximal at PZ (central parietal electrode).

The table shows the frequency of allele 1 in individuals with lifetime DSM-III-R anxiety disorders (anx) (N = 41) and no anxiety disorders (Non-Anx) (N = 282). P values are derived from a χ^2^ analysis across genotypes (2 df) and across alleles (1 df).

MAF = minor allele frequency. Rs6453267 is monomorphic in this population.

Alpha power was maximal occipitally in the U.S. Caucasians so results are presented for O1 or O2, whichever had higher power. The association results in the Plains Indians were replicated in the U.S. Caucasians (Table 5). Although the findings in this smaller dataset were not as strong, nevertheless the maximum effect size (converting back from log to absolute power) was 1.7, the same as in the Plains Indians. Allele frequencies differed considerably between the two populations (Tables 4, 5) but in both populations allele 1 was associated with increased alpha power.

**Table 5:** *CRH-BP* genotypes in U.S. Caucasians: association with resting EEG alpha power and alcohol use disorders.

SNPs	MAF	Alpha EEG power (µv^2^) at O1/O2					AUD vs No AUD			
		*CRH-BP* Genotypes			P values		Allele 1 freq		P values	
		11	12	22	Genotype	11 v 22	AUD	No AUD	Genotype	Allele
					2df	1df			2df	1df
rs32897	0.06		4.24 (0.5)	4.36 (0.5)	0.31	-	0.06	0.05	0.74	0.75
N		0	22	165						
rs6453267	0.08	4.33 (0.5)	4.36 (0.4)	4.73	0.81	-	0.95	0.91	0.46	0.22
N		157	29	1						
rs7728378	0.40	4.44 (0.5)	4.30 (0.5)	4.22 (0.4)	0.054	**0.025**	0.49	0.62	**0.004**	**0.035**
N		77	73	38						
rs1875999	0.38	4.36 (0.5)	4.37 (0.5)	4.20 (0.4)	0.24	0.092^a^	0.52	0.63	**0.046**	**0.045**
N		79	77	32						
rs10474485	0.18	4.38 (0.5)	4.30 (0.5)	4.07 (1.0)	0.23	0.137	0.73	0.82	0.216	0.078
N		125	55	6						
rs7704995	0.46	4.44 (0.5)	4.32 (0.5)	4.23 (0.4)	0.095	**0.035**	0.43	0.56	**0.005**	**0.036**
N		56	89	42						
rs1500	0.14	4.51 (0.5)	4.20 (0.5)	4.38 (0.5)	0.079	**0.034^b^**	0.20	0.13	0.169	0.121
N		5	42	140						

The table shows mean (S.D.) alpha power for individuals with each genotype (11, 12, 22). P values are derived from ANOVA; alpha power variation between genotypes (2 df) and between homozygotes (1df); ^a^11+12 v 22; ^b^12 v 22. Alpha power: log_10_ of absolute spectral power (µv^2^) in 8–13 Hz frequency band. Alpha power was maximal at O1/O2 (occipital electrodes).

The table shows the frequency of allele 1 in individuals with lifetime DSM-III-R alcohol use disorders (AUD) (N = 41) and no alcohol use disorders (Non-AUD) (N = 149).). P values are derived from a χ^2^ analysis across genotypes (2 df) and across alleles (1 df).

MAF = minor allele frequency. Rs3792738 is monomorphic in this population.

##### CRH-BP single SNP analyses: anxiety and alcohol use disorders (AUD)

In the Plains Indians, 5 SNPs within the haplotype block were significantly associated with anxiety disorders (Table 4). For rs7704995, for example, the OR for homozygote 11 vs homozygote 22 was 1.9 (1.1–3.4, 95% CI). There was no association with AUD.

In contrast, in the US Caucasians, 3 of the 4 SNPs in the haplotype block were associated with AUD but not with anxiety disorders (Table 5). For rs7704995, for example, the OR for 11 vs 22 was 1.6 (1.0–2.5, 95% CI).

##### Haplotype analyses

In the Plains Indians there were four haplotypes that accounted for 97% of the haplotype diversity (Table 6). An ANOVA revealed no global significant association between haplotypes and alpha power (F(2,589) = 2.4, p = 0.095). The two yin-yang haplotypes, 22122 and 11211, were associated with the lowest and highest alpha power respectively: p = 0.035). Haplotype frequencies differed in individuals with and without anxiety disorders: anxiety vs non anxiety: 11111: 0.12 vs 0.08; 11211: 0.23 vs 0.14; 22122: 0.65 vs 0.78 (χ^2^ = 6.4, 2df, p = 0.041).

**Table 6:** Haplotype association with alpha power in Plains Indians and U.S. Caucasians.

Plains Indians			U.S. Caucasians		
Haplotypes	Frequency	Alpha Power Mean (S.D.)	Haplotypes	Frequency	Alpha Power Mean (S.D.)
22122	0.74	4.25 (0.4)	2212	0.18	4.29 (0.4)
			2222	0.15	4.27 (0.6)
			2112	0.05	4.17 (0.5)
			2122	0.02	4.10 (0.7)
11211+11222	0.15	4.34 (0.4)	1121	0.02	3.89 (1.2)
11111	0.08	4.29 (0.3)	1111	0.44	4.39 (0.5)
			1112	0.05	4.35 (0.4)
			1211	0.04	4.42 (0.4)
			1212	0.03	4.41 (0.4)

Alpha power: log_10_ of absolute spectral power (µv^2^) in 8–13 Hz frequency band. Alpha power was maximal at PZ in Plains Indians and at O1/O2 in U.S. Caucasians. The most distal SNP, rs1500, is within the Plains Indian, but not the U.S. Caucasian, haplotype block. Plains Indians: 11222, freq 0.015, alpha power = 4.43 (0.4) was cladistically grouped with 11211.

The U.S. Caucasian haplotype block incorporated four SNPs (the most distal SNP rs1500 was not in linkage disequilibrium (LD)). There were nine haplotypes with ≥1% frequency that accounted for 98% of the haplotype diversity. Haplotypes that were cladistically derived from the 2222 ancestral haplotype were associated with low alpha power whereas haplotypes derived from the opposite 1111 ancestral haplotype were generally associated with high alpha power: F(1,374) = 6.1, p = 0.0139 (Table 6). Haplotypes derived from the 2222 ancestral haplotype were more common in alcoholics than in non-alcoholics (0.52 vs 0.38) (χ^2^ = 4.9, 1df, p = 0.027).

In summary, haplotypes 22122 in the Plains Indians and 2222-derived in the U.S. Caucasians were associated with lower alpha power in both populations, and were more abundant in U.S. Caucasian alcoholics but less abundant in Plains Indians with anxiety disorders.

## Discussion

Our study has shown that resting (eyes closed) EEG power is heritable in the Plains Indian dataset: 0.4–0.6 for alpha, 0.3 to 0.4 for theta and 0.3 for beta. Heritability is approximately the same at all scalp locations. We also demonstrated that EEG power is stable, at least over a two year period. Our results are consistent with earlier studies [15]–[19], [23] and demonstrate the stability of EEG phenotypes.

We performed multipoint linkage analysis with dense marker coverage over all autosomes: nearly 4000 unlinked SNPs at an average distance of 1 cM. The convergence of linkage peaks on chr 5 for alpha and beta EEG power produced LOD scores (3.53 and 3.48 respectively) that exceed the threshold LOD score (3.3) proposed by Lander and Kruglyak [26] for genome-wide significance in fine-mapped linkage scans of human pedigrees. Within this convergence of peaks there was also a suggestive linkage finding for theta EEG power (LOD = 2.2). Our convergent findings on chr 5 suggest a common genetic origin for alpha, beta and theta EEG power. Indeed, we expected that a substantial portion of the genetic variance would be attributable to a common source since we have shown that the genetic correlation between alpha, beta and theta power is high: approximately 0.75. This finding is supported by earlier work [17]. Further analysis revealed that *CRH-BP*, located at the apex of the convergent linkage peaks, was significantly associated with alpha power in two independent populations. In addition there was a non-significant trend for association in the same direction for theta and beta power. Although the findings were not as strong in the U.S. Caucasians as in the Plains Indians, the effect sizes were very similar (1.7 and 1.8, respectively) but the sample sizes were very different (188 and 310, respectively) suggesting that the U.S. Caucasian study was under-powered.

The aim of this study was to identify candidate genes for addiction and anxiety but also candidate genes that might be implicated in the generation of the EEG itself. Transmembrane currents originating in parietal/occipital regions for alpha and the hippocampus and limbic system for theta, are responsible for the magnitude of the recorded field. The rhythm generator for alpha originates in the thalamus [27]–[29] and in the hippocampus for theta [1] although the thalamus can act as an independent pacemaker for both alpha and theta rhythms [27]. Cholinergic and GABAergic afferents contribute to hippocampal theta activity [1]. The beta rhythm is thought to be mediated by GABA_A_ interneurons [30]. Therefore it seems likely that numerous genes, other than *CRH-BP,* may have an influence on the EEG.

In our study, *CRH-BP* variation showed the same direction of relationship to alpha power in two independent populations. Moreover, *CRH-BP* variation was significantly associated with AUD in the U.S. Caucasians and anxiety disorders in the Plains Indians. *CRH-BP* codes for a high affinity binding protein for corticotrophin releasing hormone (CRH), the primary mediator of the mammalian neuroendocrine and behavioral response to stress. In humans, CRH-BP is widely distributed throughout the body and is found in several brain regions including the cerebral cortex, the hippocampus, amygdala, lateral septal nucleus and a variety of midbrain structures [31]. Although its function in the brain is largely unknown, CRH-BP is thought to modulate CRH activity because a large proportion (65–90%) of total CRH is complexed with CRH-BP and is therefore unavailable for receptor activation [32], [33]. Centrally administered CRH increases anxiety-like behavior in rats across a wide range of paradigms [34]. Although CRH-BP deficient mice have normal baseline and stress-exposed HPA axis function they show increased anxiety-like behavior indicating an important role for CRH-BP in the CNS extra-hypothalamic stress response system [35]. CRH-BP located in the ventral tegmental area (VTA) has been shown to modulate the effects of CRH on stress-induced relapse to drug abuse [36]. Thus *CRH-BP* is a logical candidate gene for anxiety and addiction.

Although the relationship of CRH-BP to EEG is unknown, a relationship of CRH to EEG has been established in animal models, including mechanism of effect. CRH is known to increase the firing rate of LC neurons and to influence global forebrain EEG activity [37], [38]. LC activation influences the firing properties of thalamic neurons that are implicated in the generation of the alpha rhythm [27], [39] and induces fronto-cortical and hippocampal EEG changes in rats [40]. A logical possibility for the influence of *CRH-BP* on the EEG might involve CRH binding in the locus coeruleus (LC) and VTA (although *CRH-BP* mRNA has not been detected in rat LC and it is not known if it is present in human LC [31]). CRH-BP may be required for CRH to potentiate NMDAR-mediated excitatory postsynaptic currents in the VTA that herald the switch from regular firing to burst firing in dopamine neurons (41). The VTA sends dopamine containing projections to the LC that influence LC neuronal activation [42], [43]. One could therefore speculate that VTA *CRH-BP* variation might influence the resting EEG via the VTA dopamine–LC–thalamic neuronal oscillator pathway.

**Figure 3:**
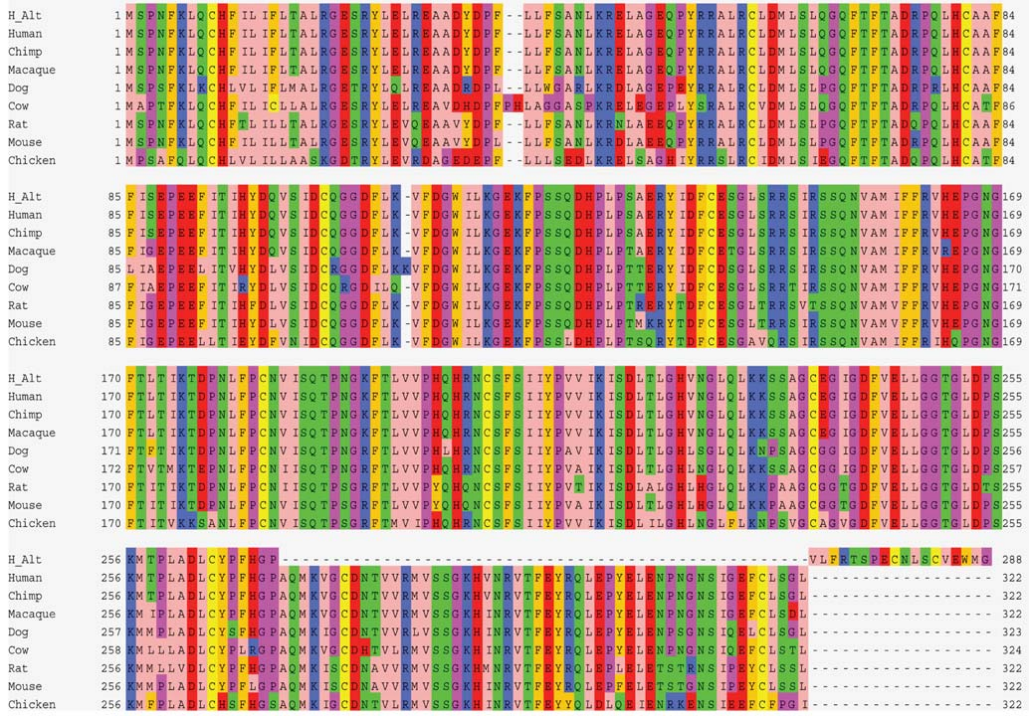
*CRH-BP* Peptide Sequence Conservation. The sequence of the human alternate isoform (H_Alt) is shown in the top line: the terminal 52 amino acids are deleted and replaced by 18 novel amino acids. Line 2 downward through line 8 shows strong sequence conservation between: human/chimp/macaque/dog/cow/rat/mouse/chicken respectively.

There is no evidence that any of the *CRH-BP* SNPs are functional. Using HapMap data it can be seen that *CRH-BP* is buffered from adjacent genes by several haplotype blocks indicating that a functional locus is likely to reside within *CRH-BP* or its environs. One mechanism for functional differences may be a second *CRH-BP* isoform that has been identified in brain. CRH-BP is highly conserved across vertebrates and in humans encodes a 322 amino acid protein that contains 5 disulphide bonds that are essential for CRH binding (Figure 3). This change in peptide sequence might affect protein folding and stability that might alter CRH binding affinity. Indeed, a C-terminus truncated peptide that shows conformational changes has been detected in the plasma of patients with inflammatory disease [44], [45]. Additionally there are regions of high conservation located within both the 3′-UTR and the introns of this novel 3′-region of the gene which may influence alternative exon usage or mRNA folding (Figure 2). The cross-species conservation is suggestive of function and may explain why we found significant associations with anxiety, alcoholism and alpha EEG power within a haplotype block that extends from intron 5 beyond the previously identified end of the gene and which encompasses the region that contains the exons encoding this novel isoform (Figure 2).

A COGA study found significant linkage of bipolar derivations of beta EEG power to a region of chr 4 (64.7–75.5 cM) that includes the GABA_A_ receptor complex (65.7–66.1 cM) (Figure S1) [24]. Using the same dataset, another study found significant linkage peaks for beta on chrs 1, 4, 5 and 15 [46]. One early study using only 95 markers found linkage for low voltage alpha EEG on chromosome 20q and noted evidence for genetic heterogeneity [47]. We did not replicate this finding made in German Caucasians. Our study also identified suggestive linkage peaks for EEG power on chr 4 but for theta and alpha and not beta, and at 40 cM–48 cM; i.e. nowhere near the GABA_A_ receptor complex. Moreover COGA's chr 5 linkage peak at 233 cM was nowhere near ours, nor did we identify linkage peaks on chrs 1 and 15. Also, COGA found no evidence for linkage for alpha or theta EEG power. Reasons for a lack of comparability between our and the COGA study may include differences in montage (we used the more common monopolar montage), derivation of EEG spectral power and dataset structure. Interestingly, a recent linkage study of nicotine addiction in an African American sample using pairwise non-parametric analysis found a chr 5 linkage peak with a LOD score of 3.04 at 95.4 cM (where alpha power in our study had a LOD score of 3.27). Multipoint non-parametric analysis identified a linkage peak with a LOD score of 2.31 at 103.5 cM (where alpha power in our study had a LOD score of 2.2) [48].

We recorded the EEG from only six electrodes because ours was an offsite study in more than 300 participants and the acquisition of quality data had to be balanced against study time. These six electrodes were selected to focus on alpha power nevertheless we found linkage peaks for beta and theta power. Although the strongest chr 5 linkage peak for alpha was at O1 the strongest association with *CRH-BP* was at PZ where EEG power was maximal in Plains Indians. In the US Caucasians EEG power was maximal at O1/O2 and was associated with *CRH-BP* variation. Finally, no corrections were made for multiple testing because EEG power at all leads was highly correlated, moreover our results were replicated in an independent dataset.

In conclusion, we and others have shown that resting EEG power is heritable and as discussed above, numerous genes are likely to contribute to variation in EEG power. So far, only one published study [25] has identified a gene (*GABRA2*) that may influence EEG power. Our linkage scan identified *CRH-BP* as a strong candidate gene and we subsequently showed that *CRH-BP* is associated with alpha power, AUD and anxiety disorders in two ethnically diverse populations. Further work needs to be done to identify functional loci for *GABRA2* and *CRH-BP*. Here, the use of a large family in an American Indian population isolate may have been important to identify linkage for a trait which may show high genetic heterogeneity. In terms of cross population validation, it is therefore of interest that our results were replicable in Caucasians.

## Materials and Methods

### There were three phases to this study

#### PHASE 1: Initial recruitment

580 men and women were recruited from a Plains Indian tribe living in rural Oklahoma. Probands were initially ascertained at random from the tribal register and the families of alcoholic probands were extended, largely coalescing into one pedigree. Written informed consent was obtained according to a human research protocol approved by the human research committee of the National Institute on Alcohol Abuse and Alcoholism (NIAAA), NIH. Ethics approval for the study was granted by the NIAAA IRB. The protocol and consent forms were also approved by the Plains Indian Tribal Council. Blind-rated DSM-III-R lifetime psychiatric diagnoses [49] were derived from the Schedule for Affective Disorders and Schizophrenia-Lifetime Version (SADS-L).

#### PHASE 2: Pilot study and EEG replication study

We re-recruited 69 individuals from the original study in order to evaluate the feasibility of running an offsite EEG study in this population and acquiring high quality data. They underwent the same procedures and satisfied the inclusion and exclusion criteria described in the Phase 3 EEG linkage study. Forty three individuals participated in both the Phase 2 and Phase 3 studies and had a repeat EEG after 2 years under the same paradigm.

#### PHASE 3: EEG linkage study

373 volunteers (215 women, 158 men) were recruited: 256 of the original Phase 1 study participants and 117 of their family members. The mean (S.D.) ages of participants were; women: 43.8 (14.7) yrs; men: 42.0 (12.7) yrs. The prevalence of DSM-III-R lifetime alcoholism was 0.75 in men and 0.46 in women. In this population the use of recreational drugs other than cannabis was rare.

Exclusion criteria included a history of severe head injury with loss of consciousness, epilepsy, seizures, stroke, brain tumors, neurological diseases, current use of psychotropic medications, chronic drug use, and those individuals unable to be off alcohol for 24 hrs without withdrawal effects. An alcohol breath test was performed at the time of the EEG recording. No participant showed a positive breathalyzer test for alcohol and none showed clinical signs of alcohol withdrawal.

#### EEG acquisition

Participants were asked to refrain from consuming alcohol and non-prescription drugs for 24 hrs prior to testing, to sleep well the night before the EEG recording, not to smoke during the procedure and not to drink more than one cup of coffee on the morning of testing.

EEG signals were recorded from a customized fitted electrode cap (Electro-Cap International Inc, Eaton, OH) with pure tin electrodes in a six channel montage: FZ (frontal-central), P3, PZ, P4 (parietal-central, left and right) and O1, O2 (occipital left and right) with reference to balanced sternovertebral electrodes. Since this study was run offsite the acquisition of quality data had to be balanced against study time. We selected these six electrode locations out of the 19 electrodes specified in the 10-20 International System because alpha power is the predominant EEG waveform in the relaxed, alert individual and alpha power is maximal posteriorly. The FZ electrode was selected to allow acquisition of frontal beta and theta EEG. Additional electrodes were applied below and lateral to the left eye to monitor electro-oculographic artifacts. Electrode impedance was kept below 5 kohms.

Each subject was seated in a chair in a darkened room. External sounds were dampened by foam insert earphones and by continuous white noise. Resting EEG was recorded continuously for 3 min with eyes open and then for 3 min with eyes closed. Every effort was made to ensure that the participant was relaxed, but alert. An observer monitored the participant and EEG tracing for signs of drowsiness (alpha wave drop-out) or movement. The session was repeated if substantial drowsiness, sleep or movement was detected. Only the eyes-closed EEG was used for these analyses.

The channels were connected to a Biopac MP100 system using ECG100C digital biodifferential amplifiers. Waveforms were amplified with a gain of 10,000 with a bandpass of 0.1–100 Hz, with a common mode rejection ratio of 100 dB min at 1000 Mohm. FPZ was used as the ground electrode.

#### EEG Analysis

EEG signals were continuously digitized throughout each recording session at a sampling rate of 200 Hz. Data were digitally filtered offline using a 299-tap, finite-impulse response, low pass algorithm with a cut-off of 30 Hz (Hamming window, 10 dB down). Quantitative spectral analysis was then performed [50]. To minimize any non-optimized common-mode rejection artifacts contributing to baseline offset (i.e., any twos-complement quantization plateau introduced from the AC amplifiers), a running mean was uniformly subtracted from the signal [51], [52]. The mean subtraction was a simple offline DC offset correction, and not a boxcar filter, with its inherent side effects [53]. Data records were partitioned into consecutive 512-point subunits (2.56 sec each). These partitions were then autoregressively filtered to remove low frequency subharmonics and were tapered using a cosine-bell window [50]. Fast Fourier transformation of these filtered partitions yielded power spectrum estimates in 0.39-Hz steps.

For the purposes of deletion of drowsiness or sleep a temporary data file was first constructed for each EEG record using a digital, finite-impulse response, bandpass filter with cutoffs at 2.5 to 30 Hz. A computer monitor displayed consecutive 5 sec intervals of EEG waveforms with gridlines at 1 sec intervals. All three minutes of eyes-closed EEG was scanned in this way and epochs containing artifacts, drowsiness or sleep were deleted. Almost all subjects showed 100 sec or more of acceptable EEG recordings. The minimum was twenty five 2.56 sec epochs. Data from acceptable epochs was averaged to yield a composite spectrum spanning three frequency domains: 3–8 Hz, 8–13 Hz and 13–30 Hz. We excluded the delta band because of the likelihood of large measurement error due, for example, to residual eye movement artifacts as has been found in other studies [17]. The absolute spectral power in each of the three frequency bands at each of the six electrode locations was log transformed to normalize the distribution. The EEG data from 9 participants was excluded because of poor quality and therefore the EEG dataset comprised 364 individuals.

#### The U.S. Caucasian replication EEG dataset

The EEG phenotype-genotype association results were replicated in a second EEG dataset; U.S. Caucasians recruited in Bethesda, MD: 104 women and 84 men. The mean (S.D.) ages of participants were; women: 43.2 (15.8) yrs; men: 40.8 (16.9) yrs. This EEG dataset has been fully described elsewhere [9]. The paradigm for EEG acquisition and analysis was the same as described above for the Plains Indians except that in the Bethesda sample data were recorded from gold-plated electrodes applied to 19 scalp sites identified in the International 10-20 System and at two mastoid sites, all with reference to balanced sternovertebral electrodes.

#### Pedigree structure

Pedigrees were derived from the 580 tribal members recruited in phase 1 and the 117 additional participants recruited in phase 3. Pedigrees were constructed through a lengthy process of consultation and discussion with senior tribal members. In addition, the DNA samples of phase 3 participants were genotyped for 10 short tandem repeat markers to correct for Mendelian inconsistencies. PedCheck [54] was used as a second check for Mendelian inconsistent genotyping data. Most tribal members belonged to one large pedigree (n = 1004). In addition 101 tribal members were distributed in five other pedigrees (n = 14–33).

DNA was available for 353 Plains Indians. Six individuals who were misgendered/mixed identity were excluded. Nineteen samples failed to genotype. Therefore data from 328 DNAs were used in the transmission and linkage analyses after confirmation. Genotypes plus EEG data were available for 266 Plains Indians: 249 from the one large pedigree and 17 from the 5 small pedigrees (2–5 per pedigree).

We ran LOKI, a linkage analysis package for quantitative traits within large and complex pedigrees which uses Markov chain Monte Carlo techniques to calculate kinship coefficients between any two individuals (www.helix.nih.gov/apps/bioinfo/loki.html). The numbers of relative pairs calculated using the kinship coefficient were as follows: 1^st^ degree: 384; 2^nd^ degree: 349; 3^rd^ degree: 495; 4^th^ degree: 547; <5% related: 1466. The percentage of genetic identity shared between any two individuals in the pedigrees through common descent was calculated for all possible pairs (related and unrelated) using S.A.G.E. (Case Western Reserve University). The average sharing of descent was 0.3%, which is less than the degree of relationship between third cousins.

#### Genotyping

Samples were genotyped with the 5861 SNP Illumina Linkage IV-B panel. Genotyping was performed on the Illumina GoldenGate platform under contract at Illumina. Genotyping reproducibility was 99.96% and the genotype call rate was 99.95%. Non-informative (monomorphic) markers were deleted from the analyses.

Linkage disequilibrium (LD) among markers can induce false-positive evidence of linkage in multipoint linkage analyses. Some of the 5861 SNPs were in LD. Kinship coefficient calculations by LOKI identified 42 completely unrelated genotyped individuals within the pedigrees. HAPLOVIEW was used to calculate marker LD in these 42 individuals. Markers in LD (on average 34% of markers per chromosome) were excluded according to HAPLOVIEW criteria including D′>0.5, with the exception of a single marker within a defined LD group (Figure S2). The resulting 3878 unlinked markers were used in the linkage analyses. The mean distance between unlinked markers was 1 cM, the minimum distance permitted by SOLAR (sequential oligogenic linkage analysis routine) [55]. Genetic map positions (cM) were derived from deCODE (Genethon, Marshfield). The logarithm of the odds (LOD) scores were computed from the 3878 markers not in LD using SOLAR.

#### Linkage analysis

Multipoint variance component linkage analyses were conducted using SOLAR version 4.0.7. SOLAR is a computer package based on the variance-component method that performs linkage analyses for quantitative traits in pedigrees of arbitrary size and complexity, and was applicable to this study's large, complex pedigree with inbreeding loops and multiple matings. The variance component method is used both for localization of quantitative trait loci (QTLs) and to estimate the proportion of phenotypic variance that can be explained by a specific QTL in the population. SOLAR is also implemented to perform linkage to discrete traits by using a threshold model. Due to the size of the pedigree, LOKI was used to compute the SOLAR-ready Multipoint Identity by Descent matrices.

#### Heritability of phenotypic traits; genetic correlation between traits

SOLAR version 4.0.7 was used to fit a variance components model for estimating heritability. In SOLAR, maximum likelihood estimation is applied to a mixed-effects model that includes fixed covariate effects, additive genetic effects and residual error. Heritability is calculated as the proportion of phenotypic variance explained by additive genetic effects while accounting for covariates. Z scores were computed for the log_10_ EEG power variables. Age was a significant covariate for alpha power at all leads contributing between 0.009 (O1) to 0.035 of the variance at PZ. After transformation by Z scores and covariate adjustment for age and sex the residual kurtosis was normal for alpha power but slightly elevated (>0.5) for theta (approximately 1 for all leads except O1 (2.2)) and for beta (0.8–1.0). The high kurtosis (2.2) for theta power at O1 was corrected by inverse normalization using SOLAR version 4.1.0.

The bivariate models implemented in SOLAR version 4.1.0 were used to test for genetic correlations between EEG power in different frequency bands at PZ where alpha power was maximal. SOLAR can apportion the phenotypic correlation between two continuous traits into genetic and environmental correlations using multivariate polygenic analysis.

#### Verification of linkage results by quantitative trait simulation

Empirical p values for the linkage peaks were obtained by performing 10,000 simulations using SOLAR. In SOLAR, simulations are performed with the quantitative traits while the genotypes are kept constant. A model is specified which describes the phenotypic distribution (mean and variance), the genetic components of the trait (heritability due to an underlying QTL and that attributable to polygenes) and the effects of any covariates (sex and age).

#### Candidate gene selection

All genes under linkage peaks with a LOD score ≥2 were located by converting genetic map (deCODE) cM numbers to human genome chromosome physical positions and then using the NCBI database (Build 36). Candidate genes for resting EEG, anxiety and addiction were identified.

#### Candidate gene association study: *CRH-BP*

A 1,536 tag SNP addiction array has been developed within the Laboratory of Neurogenetics, NIAAA, allowing for the analysis of 130 candidate genes for the addictions and for 186 ancestry informative markers [56]. Genotypes for 8 SNPs across the corticotropin releasing hormone binding protein (*CRH-BP*) gene were available from the addiction array for 310 Plains Indians with EEG data (Table 4) and for 186–188 of the U.S. Caucasians (Table 5). As described above, we calculated that the average percentage of genetic identity shared between any two individuals through common descent for all possible pairs (related and unrelated) was 0.3%, which is less than the degree of relationship between third cousins. Therefore ANOVA was performed to test the association between *CRH-BP* genotypes and EEG power. The significant p values were confirmed by 10,000 simulations using SIMPED [57]. With this program genotypes can be generated conditional on the user-specified frequencies using the gene-drop method within pedigrees of virtually any size and complexity. Since the four significant SNPs were in allelic identity, association analysis (t-test between the two homozygous genotypes) was computed for the simulated SNP rs7704995 and a null distribution for the t statistic was generated. This was subsequently used to determine the empirical p value.

##### CRH-BP haplotype analyses

Haplotype frequencies were estimated using a Bayesian approach implemented with PHASE [58]. Haploview version 2.04 Software (Whitehead Institute for Biomedical Research, USA) was used to produce LD matrices.

#### Assessment of population stratification using ancestry informative markers (AIMS)

The Plains Indian and U.S. Caucasian samples were genotyped for 186 ancestry markers that were on the addiction array described above [56]. The AIMs were also genotyped in 1051 individuals from 51 worldwide populations represented in the HGDP-CEPH Human Genome Diversity Cell Line Panel (http://www.cephb.fr/HGDP-CEPH-Panel). PHASE *Structure 2.2* (http://pritch.bsd.uchicago.edu/software.html) was run simultaneously using the AIMS genotypes from our two samples and the 51 CEPH populations to identify population substructure and compute individual ethnic factor scores. This “anchored” approach showed that a seven factor solution was optimal. To evaluate the potential impact of ethnic substructure on our association results, individual alpha power was correlated with individual ethnic factor scores using the Spearman ρ correlation. There were no significant correlations with any of the seven ethnic factors (U.S. Caucasians p = 0.21–0.99; Plains Indians p = 0.3–0.94). These results indicate that ethnic substructure is unlikely to have an impact on the association analyses.

## Supporting Information

Materials S1 Further results from the genome linkage scan. Presentation of results showing suggestive linkage peaks and discussion of potential candidate genes.
(0.04 MB DOC)

Figure S1 Linkage Peaks for Resting EEG Power on Chromosomes 22, 4, 10 and 11 and Locations of Candidate Genes.
(0.08 MB TIF)

Figure S2 Illustration of Method for Eliminating SNPs that are in Linkage Disequilibrium.
(5.99 MB EPS)
