# Supplementary material for: Common Genetic Origins for EEG, Alcoholism and Anxiety: The Role of CRH-BP
Source: PLoS One. 2008 Oct 31;3(10):e3620. doi: 10.1371/journal.pone.0003620 (PMC2575401; doi:10.1371/journal.pone.0003620)
Supplement: Materials S1 — Further results from the genome linkage scan. Presentation of results showing suggestive linkage peaks and discussion of potential candidate genes. (0.04 MB DOC) [file pone.0003620.s001.doc]

**SUPPLEMENTARY MATERIALS**

**Common Genetic Origins for EEG, Alcoholism and Anxiety: the Role of *CRH-BP***

Mary-Anne Enoch ^1^, Pei-Hong Shen^1^, Francesca Ducci^1^, Qiaoping Yuan^1^, Jixia Liu^1^, Kenneth V. White^1^, Bernard Albaugh^2^, Colin A. Hodgkinson^1^ and David Goldman^1^

^1^Laboratory of Neurogenetics, National Institute on Alcohol Abuse and Alcoholism, NIH, Bethesda, MD 20892, USA.

^2^ Center for Human Behavior Studies, Inc, Weatherford, OK 73096, USA.

Corresponding author:

Mary-Anne Enoch M.D.

NIH/NIAAA/DICBR/LNG

5625 Fishers Lane, Room 3S32, MSC 9412

Bethesda, MD 20892-9412

Tel: 301-496-2727

Fax: 301-480-2839

email: [maenoch@niaaa.nih.gov](mailto:maenoch@niaaa.nih.gov)

**Results from the whole genome linkage scan**

***Suggestive Linkage (LOD > 2.0):***

We found suggestive evidence for linkage that was specific to particular EEG frequency bands: theta on chr 22 (LOD = 3.2) and on chr 4 (LOD = 2.5); alpha on chr 4 (LOD = 2.4) and on chr 11 (LOD = 2.2) and beta on chr 10 (LOD = 2.5) and on chr 2 (LOD = 2.1).

***Chromosome 22***

There was a linkage peak for theta EEG power at O1 with a LOD score of 3.2 (empirical p = 0.0001) at 20 cM (Table 2, Figure 2).

*Candidate genes*

There are 325 genes in the region of the peak with a LOD score of > 2.0 (4 – 26 cM; 16.5 – 25.3 Mbp). The majority of these are immunoglobulin genes (20.7 – 21.5 Mbp). Based upon gene function there were three likely candidate genes in the region, all located at sites with a maximum LOD score of 2.3: adenosine A2a receptor (*ADORA2A,* MIM 102776) (19cM); mitogen activated protein kinase 1 (*MAPK1,* MIM 176948) (14 cM) and catechol –O-methyltransferase (*COMT,* MIM 116790) (10 cM).

***Chromosome 4***

There were linkage peaks for theta EEG power at O1 (LOD = 2.5 at 40 cM) and alpha power at O2 (LOD = 2.4 at 48 cM) (Table 2, Figure 2).

*Candidate genes*

There were 56 genes in region of the linkage peak for theta EEG power at O1 that had a LOD score of > 1.5 (30 – 47 cM; 15.2 – 27.4 Mbp). The gene for the cholecystokinin A receptor (*CCKAR,* MIM 118444) is located within the peak at 45.5 CM, with a LOD score of 1.7. There were 52 genes in the overlapping peak region for alpha power at O2. The only likely candidate gene at this location is *CCKAR*.

***Chromosomes 10 and 2***

There was a linkage peak for beta power at O1 at 110 cM, LOD = 2.5 (Figure 2).

*Candidate genes*

There were 89 genes in the region (103 – 113 cM, 83.8 – 95.4 Mbp) under the peak with a LOD score of > 1.5. Candidate genes include the serotonin receptor 7 (*HTR7,* MIM 182137) (111 cM) and glutamate dehydrogenase 1 (*GLUD1,* MIM 138130) at 107.5 cM.

There was also a linkage peak for beta power at O1 on chr 2 (244 cM) with a LOD score of 2.1. A candidate gene in this area is serotonin receptor 2B (*HTR2B,* MIM 601122) at 238 cM.

***Chromosome 11***

There was a linkage peak for alpha power at P3 (LOD = 2.2), 114 cM (Figure 2).

*Candidate genes*

There were 66 genes in the region (109 – 117 cM; 109.7 – 116.3 Mbp) under the peak for alpha power at LOD > 1.5). There were four strong candidate genes located close together near the apex of the linkage peak (114 cM): serotonin receptors 3A and 3B (*HTR3A* [MIM 182139] and *HTR3B* [MIM 604654]) at 113.2 cM, dopamine receptor 2 (*DRD2*, MIM 126450) and ankyrin repeat and kinase domain containing 1 (*ANKKI*, MIM 608774) at 112.7 cM.

**Candidate genes for further study**

We identified other candidate genes that will be explored in later analyses. *HOMER1*, located close to the linkage peak on chr 5, is a synaptic scaffolding protein that regulates signal transduction and synaptogenesis; preclinical studies indicate that it may have a role in addiction, anxiety and epilepsy [S1]. *ADORA2A*, a major target of caffeine that has recently been implicated in alcoholism [S2] was located close to the apex of the theta linkage peak on chr 22 (LOD = 3.2) whereas *COMT*, previously associated with the low voltage alpha EEG phenotype [S3] was located more peripherally. *CCKAR*, located at the apex of the linkage peaks for alpha and theta power on chr 4 (LOD = 2.5) is a G-protein-coupled receptor that regulates the release of beta endorphin and dopamine in the CNS and is implicated in stress. Linkage peaks identified several serotonin receptors as candidate genes: *HTR7* (chr 10), *HTR3A* and *HTR3B* (chr 11), *HTR2B* (chr 2) and *HTR1A* (chr 5). *DRD2* and *ANKKI* were also located at the apex of the peak on chr 11*.*

**REFERENCES**

S1. Szumlinski KK, Kalivas PW, Worley PF (2006) Homer proteins: implications for neuropsychiatric disorders. Curr Opin Neurobiol 16: 251-257.

S2. Thorsell A, Johnson J, Heilig M (2007) Effect of the adenosine A2a receptor antagonist 3,7-dimethyl-propargylxanthine on anxiety-like and depression-like behavior and alcohol consumption in Wistar Rats. Alcohol Clin Exp Res 31: 1302-1307.

S3. Enoch MA, Xu K, Ferro E, Harris CR, Goldman D (2003) Genetic origins of anxiety in women; a role for a functional COMT polymorphism. Psychiatr Genet 13: 33-41.

**FIGURE S1**

**Linkage Peaks for Resting EEG Power on Chromosomes 22, 4, 10 and 11 and Locations of Candidate Genes.**

EEG power frequency bands: theta (3 – 8 Hz), alpha (8 – 13 Hz) and beta (13 – 30 Hz).

O1, O2 = occipital electrodes; P3, PZ, P4 = parietal electrodes. FZ = frontal-central electrode. GABA_A_ receptor complex: gene cluster for GABAA receptors. *CCKAR* = cholecystokinin A receptor gene; *HTR7* = serotonin receptor 7 gene; *HTR3A* and *HTR3B* = serotonin receptor 3A and 3B genes. *DRD2* = dopamine receptor 2 gene. *ANKKI* = ankyrin repeat and kinase domain containing 1 gene.

**FIGURE S2**

**Illustration of Method for Eliminating SNPs that are in Linkage Disequilibrium**

Red diamonds indicate linkage disequilibrium (LD) (D’ > 0.5) between SNPs. All markers in LD were eliminated (N = 1983) resulting in 3878 unlinked SNPs that were used in the linkage analyses.
