# Supplementary figures and images for: Common Genetic Origins for EEG, Alcoholism and Anxiety: The Role of CRH-BP
Source: PLoS One. 2008 Oct 31;3(10):e3620. doi: 10.1371/journal.pone.0003620 (PMC2575401; doi:10.1371/journal.pone.0003620)

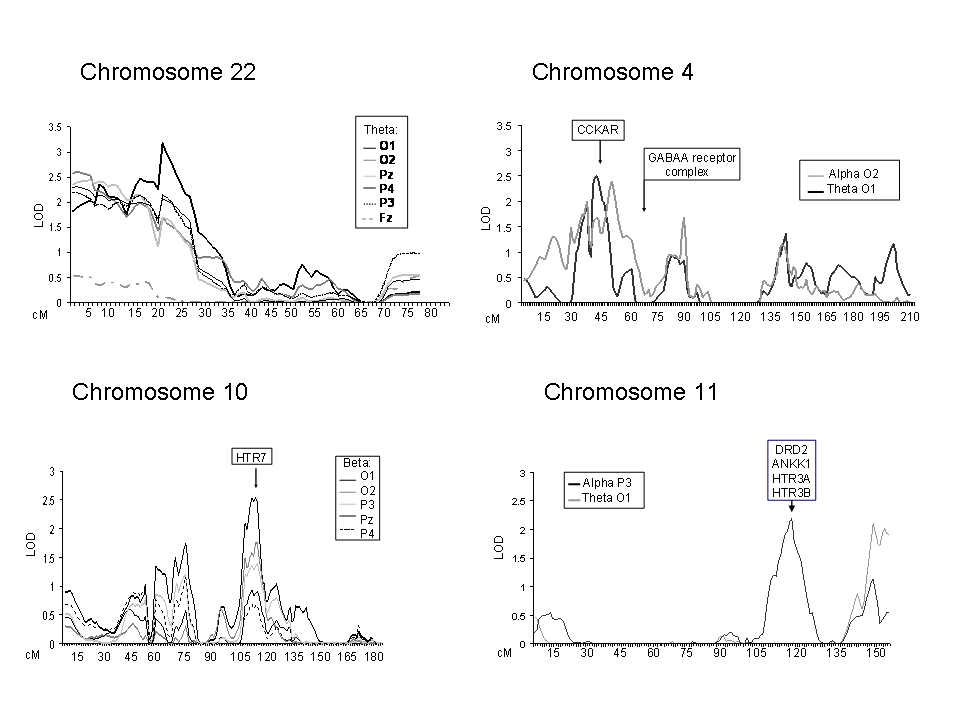

Supplement: Figure S1 — Linkage Peaks for Resting EEG Power on Chromosomes 22, 4, 10 and 11 and Locations of Candidate Genes. (0.08 MB TIF) [file pone.0003620.s002.tif]
